# Crystal structure of *N*,*N*,*N*-tri­ethyl­hydroxyl­ammonium chloride

**DOI:** 10.1107/S2056989016016169

**Published:** 2016-10-21

**Authors:** Boris B. Averkiev, Bianca C. Valencia, Yulia A. Getmanenko, Tatiana V. Timofeeva

**Affiliations:** a Department of Chemistry, New Mexico Highlands University, Las Vegas, NM 87701, USA; b ITMO University, 49 Kronverkskiy Prospekt, Saint Petersburg, 197101 , Russian Federation

**Keywords:** crystal structure, hydrogen bonding, C—H⋯Cl contacts

## Abstract

In the title mol­ecular salt, C_6_H_16_NO^+^·Cl^−^, two of the C—C—N—O groups in the cation adopt a *gauche* conformation [torsion angles = 62.86 (11) and −54.95 (13)°] and one an *anti* conformation [−177.82 (10)°. The cation and anion are linked by an O—H⋯Cl hydrogen bond. The extended structure displays C—H⋯Cl and C—H⋯O hydrogen bonds, resulting in layers lying parallel to the (100) plane: further C—H⋯Cl contacts connect the sheets into a three-dimensional network.

## Abstract 2

The cation and anion in *N*,*N*,*N*-tri­ethyl­hydroxyl­ammonium chloride are linked by an O—H⋯Cl hydrogen bond. The extended structure displays C—H⋯Cl and C—H⋯O hydrogen bonds, resulting in layers lying parallel to the (100) plane: further C—H⋯Cl contacts connect the sheets into a three-dimensional network.

## Chemical context   

Tri­ethyl­amine is often used to treat silica gel with the goal of reducing the acidity of the stationary phase during column chromatography purification. In a typical procedure, an eluant is mixed with tri­ethyl­amine (1–3%), and this solvent mixture is used to prepare the silica gel slurry for manually packed columns. While the effect of the tri­ethyl­amine on silica gel is known, no specific details could be found about the structural transformation of tri­ethyl­amine itself. This work presents the result of the column chromatography purification of a di­thia­zolo[4,5-*a*:5′,4′-*c*]phenazine derivative using a di­chloro­methane:ethyl acetate mixture as eluant. Unexpectedly, the crystals obtained after slow solvent evaporation from an ‘empty’ fraction were identified as the title mol­ecular salt, *N*,*N*,*N*-tri­ethyl­hydroxyl­ammonium chloride, **1**.
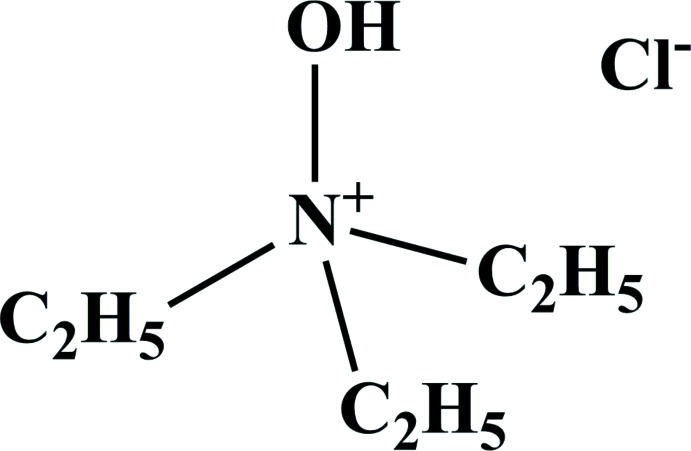


## Structural commentary   

**Figure 1:**
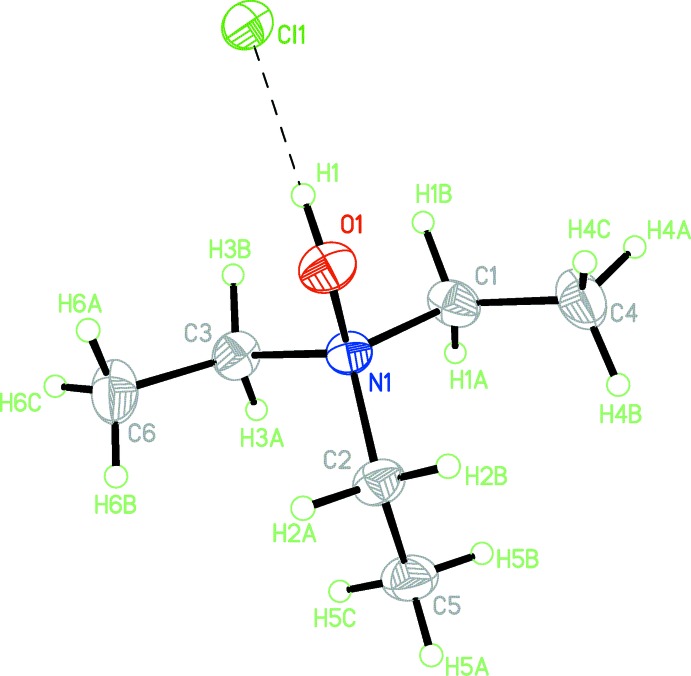
The mol­ecular structure of **1**, with displacement ellipsoids drawn at the 50% probability level. The O1—H1⋯Cl1 hydrogen bond is shown as a dashed line (see Table 1 ▸).

The mol­ecular structure of **1** is presented in Fig. 1 ▸. The C—N bond lengths [1.5090 (13)–1.5148 (13) Å] and the N—O bond length [1.4218 (11) Å] are in good agreement with mean reported geometries for 79 entries from the Cambridge Structural Database (CSD; Groom *et al.*, 2016 ▸) containing the C_3_N—O—*R* (*R* = C, H) fragment: C—N 1.51 (3) Å and N—O 1.42 (2) Å and comparable to the analogous data in a closely related compound, *N*,*N*,*N*-tri­methyl­hydroxyl­ammonium chloride, **2** (1.488–1.489 Å for the N—C bonds and 1.429 Å for the N—O bond) (Jiang *et al.*, 2004 ▸; Rérat, 1960 ▸; Caron & Donohue, 1962 ▸). The hydroxyl hydrogen atom H1 participates in a strong hydrogen bond with the adjacent chloride anion (Table 1 ▸), which is also observed for **2**.

## Supra­molecular features   

**Figure 2:**
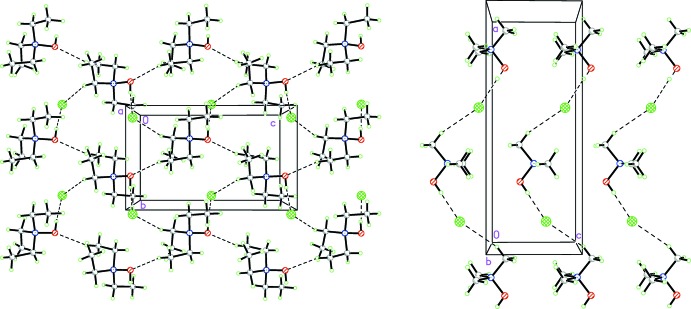
Layers in the crystal structures of (left) **1** and (right) **2**.

The O1—H1⋯Cl1 and C1—H1*A*⋯Cl1 hydrogen bonds assemble the constituent ions into spiral chains around 2_1_ axes. These chains are connected by C5—H5*B*⋯O1 hydrogen bonds into sheets lying parallel to the (100) plane (Fig. 2 ▸). There are four weak C—H⋯Cl contacts in the structure. The C2—H3*B*⋯Cl1 contact reinforces the O—H⋯Cl hydrogen bond; the C3—H3*A*⋯Cl1 hydrogen bond connects mol­ecules within a sheet, while the C2—H2*A*⋯Cl1 and C4—H4*A*⋯Cl1 contacts connect the ions between the (100) sheets.

For comparison, the crystal packing of **2** is rather different. The cations in **2** lie on mirror planes and are arranged into chains along the [100] direction, being linked by O1–H1⋯Cl1 and C2–H5⋯Cl1 hydrogen bonds. The mol­ecules in the chain are symmetrically related by a glide plane and C1—H2⋯Cl1 hydrogen bonds connect the chains into three-dimensional network. It is noteworthy that the oxygen atom does not participate as a proton acceptor in hydrogen bonding.

## Database survey   

A search of the Cambridge Structural Database (Groom *et al.*, 2016 ▸) revealed 221 crystal structures containing the C_3_N–O fragment: 144 of them contain a C_3_N^+^—O^−^ fragment and 79 a C_3_N–O–*R* fragment (*R* = C, H). While the additional connection of the oxygen atom increases the N—O bond length from 1.393 (18) to 1.42 (2) Å, the C—N bond lengths are not affected and remain at 1.51 (3) Å value. 31 structures in the CSD are salts of the C_3_N^+^–OH cation. In eight of them, the anion is Cl^−^, of which seven feature an O—H⋯Cl hydrogen bond (the O⋯Cl distance varies from 2.872 to 3.010 Å). The exception is the crystal structure of (1*S*,5*S*)-geneseroline hydro­chloride monohydrate (refcode VAVZUN), in which the solvent water mol­ecule accepts an O—H⋯O hydrogen bond from the C_3_N^+^–OH group.

## Synthesis and crystallization   

During the column chromatography purification of the di­thia­zolo[4,5-*a*:5′,4′-*c*]phenazine derivative using di­chloro­methane–ethyl ­acetate as eluant and Alfa–Aesar silica gel (stock # 42570; lot # K03T015; case # 632131-67-4) treated with tri­ethyl­amine, a fraction containing a trace amount of the desired product was left over several days until compete evaporation of the solvents led to the formation of colourless plates of the title compound. Unexpectedly, the crystals were identified as *N*,*N*,*N*-tri­ethyl­hydroxyl­ammonium chloride; di­chloro­methane was probably the source of the chloride anion.

## Refinement   

Crystal data, data collection and structure refinement details are summarized in Table 2 ▸. H atoms were all located in difference Fourier map and refined isotropically.

## Supplementary Material

Crystal structure: contains datablock(s) I. DOI: 10.1107/S2056989016016169/hb7617sup1.cif

Structure factors: contains datablock(s) I. DOI: 10.1107/S2056989016016169/hb7617Isup2.hkl

Supporting information file. DOI: 10.1107/S2056989016016169/hb7617Isup3.cml

CCDC reference: 1509423

Additional supporting information: crystallographic information; 3D view; checkCIF report

## Figures and Tables

**Table 1:** Hydrogen-bond geometry (Å, °)

*D*—H⋯*A*	*D*—H	H⋯*A*	*D*⋯*A*	*D*—H⋯*A*
O1—H1⋯Cl1	0.87 (2)	2.06 (2)	2.9330 (12)	175 (2)
C1—H1*A*⋯Cl1^i^	0.934 (18)	2.888 (19)	3.7786 (15)	159.8 (15)
C2—H2*A*⋯Cl1^ii^	0.955 (18)	2.943 (17)	3.6859 (16)	135.6 (14)
C3—H3*B*⋯Cl1	0.977 (19)	2.911 (19)	3.6203 (16)	130.3 (14)
C3—H3*A*⋯Cl1^i^	0.93 (2)	2.93 (2)	3.7740 (19)	150.7 (14)
C4—H4*A*⋯Cl1^iii^	1.02 (4)	2.98 (4)	3.9913 (18)	172 (2)
C5—H5*B*⋯O1^iv^	0.97 (3)	2.50 (3)	3.4359 (18)	163 (2)

Symmetry codes: (i) 
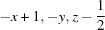
; (ii) 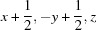; (iii) 
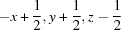
; (iv) 
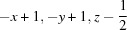
.

**Table 2:** Experimental details

Crystal data	
Chemical formula	C_6_H_16_NO^+^·Cl^−^
*M*_r_	153.65
Crystal system, space group	Orthorhombic, *P* *n* *a*2_1_
Temperature (K)	215
*a*, *b*, *c* (Å)	12.816 (5), 6.371 (3), 10.439 (4)
*V* (Å^3^)	852.3 (6)
*Z*	4
Radiation type	Mo *K*α
μ (mm^−1^)	0.38
Crystal size (mm)	0.40 × 0.20 × 0.05

Data collection	
Diffractometer	Bruker APEXII CCD
Absorption correction	Multi-scan (*SADABS*; Bruker, 2008 ▸)
*T*_min_, *T*_max_	0.667, 0.746
No. of measured, independent and observed [*I* > 2σ(*I*)] reflections	12103, 2484, 2447
*R*_int_	0.025
(sin θ/λ)_max_ (Å^−1^)	0.703

Refinement	
*R*[*F* ^2^ > 2σ(*F* ^2^)], *wR*(*F* ^2^), *S*	0.021, 0.056, 1.04
No. of reflections	2484
No. of parameters	146
No. of restraints	1
H-atom treatment	All H-atom parameters refined
Δρ_max_, Δρ_min_ (e Å^−3^)	0.15, −0.14
Absolute structure	Flack *x* determined using 1146 quotients [(*I* ^+^)−(*I* ^−^)]/[(*I* ^+^)+(*I* ^−^)] (Parsons et al., 2013 ▸)
Absolute structure parameter	0.046 (15)

Computer programs: *APEX2* and *SAINT* (Bruker, 2008 ▸), *SHELXS97* and *SHELXTL*(Sheldrick, 2008 ▸) and *SHELXL2014* (Sheldrick, 2015 ▸).

## supplementary crystallographic information

### Crystal data

**Table:**

C_6_H_16_NO^+^·Cl^−^	*D*_x_ = 1.197 Mg m^−^^3^
*M**_r_* = 153.65	Mo *K*α radiation, λ = 0.71073 Å
Orthorhombic, *P**n**a*2_1_	Cell parameters from 2230 reflections
*a* = 12.816 (5) Å	θ = 3.6–32.3°
*b* = 6.371 (3) Å	µ = 0.38 mm^−^^1^
*c* = 10.439 (4) Å	*T* = 215 K
*V* = 852.3 (6) Å^3^	Plate, colorless
*Z* = 4	0.40 × 0.20 × 0.05 mm
*F*(000) = 336	

### Data collection

**Table:**

Bruker APEXII CCD diffractometer	2447 reflections with *I* > 2σ(*I*)
φ and ω scans	*R*_int_ = 0.025
Absorption correction: multi-scan (SADABS; Bruker, 2008)	θ_max_ = 30.0°, θ_min_ = 3.2°
*T*_min_ = 0.667, *T*_max_ = 0.746	*h* = −18→18
12103 measured reflections	*k* = −8→8
2484 independent reflections	*l* = −14→14

### Refinement

**Table:**

Refinement on *F*^2^	Hydrogen site location: difference Fourier map
Least-squares matrix: full	All H-atom parameters refined
*R*[*F*^2^ > 2σ(*F*^2^)] = 0.021	*w* = 1/[σ^2^(*F*_o_^2^) + (0.0429*P*)^2^ + 0.0129*P*] where *P* = (*F*_o_^2^ + 2*F*_c_^2^)/3
*wR*(*F*^2^) = 0.056	(Δ/σ)_max_ = 0.034
*S* = 1.04	Δρ_max_ = 0.15 e Å^−^^3^
2484 reflections	Δρ_min_ = −0.14 e Å^−^^3^
146 parameters	Absolute structure: Flack *x* determined using 1146 quotients [(*I*^+^)-(*I*^-^)]/[(*I*^+^)+(*I*^-^)] (Parsons et al., 2013)
1 restraint	Absolute structure parameter: 0.046 (15)

### Special details

**Table:**

Geometry. All esds (except the esd in the dihedral angle between two l.s. planes) are estimated using the full covariance matrix. The cell esds are taken into account individually in the estimation of esds in distances, angles and torsion angles; correlations between esds in cell parameters are only used when they are defined by crystal symmetry. An approximate (isotropic) treatment of cell esds is used for estimating esds involving l.s. planes.

### Fractional atomic coordinates and isotropic or equivalent isotropic displacement parameters (Å^2^)

**Table:**

	*x*	*y*	*z*	*U*_iso_*/*U*_eq_	
Cl1	0.35543 (2)	−0.08505 (4)	0.50629 (5)	0.03438 (9)	
O1	0.47318 (6)	0.30749 (12)	0.48070 (7)	0.02789 (17)	
H1	0.4399 (16)	0.188 (4)	0.484 (3)	0.060 (6)*	
N1	0.53046 (7)	0.29675 (12)	0.36425 (9)	0.02182 (16)	
C1	0.45457 (9)	0.28944 (18)	0.25337 (10)	0.0299 (2)	
H1B	0.4138 (19)	0.155 (4)	0.269 (2)	0.054 (5)*	
H1A	0.4948 (15)	0.269 (3)	0.1798 (16)	0.031 (4)*	
C2	0.59511 (9)	0.49467 (16)	0.36668 (10)	0.0269 (2)	
H2B	0.5464 (16)	0.599 (3)	0.380 (2)	0.038 (5)*	
H2A	0.6374 (13)	0.486 (3)	0.4418 (18)	0.028 (4)*	
C3	0.59581 (10)	0.09863 (15)	0.36273 (11)	0.0278 (2)	
H3B	0.5446 (14)	−0.015 (3)	0.358 (2)	0.038 (4)*	
H3A	0.6338 (13)	0.106 (3)	0.287 (2)	0.030 (4)*	
C4	0.38747 (11)	0.4836 (3)	0.24189 (13)	0.0404 (3)	
H4C	0.3582 (16)	0.529 (4)	0.320 (2)	0.049 (6)*	
H4B	0.4278 (16)	0.602 (3)	0.207 (2)	0.040 (5)*	
H4A	0.328 (3)	0.450 (5)	0.181 (4)	0.086 (10)*	
C5	0.66019 (11)	0.5267 (2)	0.24769 (13)	0.0347 (2)	
H5C	0.712 (2)	0.420 (4)	0.240 (3)	0.077 (8)*	
H5B	0.616 (2)	0.543 (4)	0.173 (3)	0.064 (7)*	
H5A	0.6950 (19)	0.650 (4)	0.252 (2)	0.060 (6)*	
C6	0.66688 (14)	0.0771 (2)	0.47737 (14)	0.0392 (3)	
H6C	0.7011 (19)	−0.051 (4)	0.471 (2)	0.054 (6)*	
H6B	0.7175 (18)	0.182 (4)	0.478 (3)	0.066 (7)*	
H6A	0.6251 (18)	0.078 (3)	0.562 (3)	0.044 (6)*	

### Atomic displacement parameters (Å^2^)

**Table:**

	*U*^11^	*U*^22^	*U*^33^	*U*^12^	*U*^13^	*U*^23^
Cl1	0.03250 (14)	0.03626 (14)	0.03439 (14)	−0.00604 (8)	−0.00249 (12)	0.00905 (11)
O1	0.0323 (4)	0.0302 (4)	0.0212 (4)	−0.0008 (3)	0.0078 (3)	−0.0021 (2)
N1	0.0231 (4)	0.0232 (3)	0.0192 (3)	−0.0007 (3)	0.0026 (3)	−0.0014 (3)
C1	0.0257 (4)	0.0417 (6)	0.0222 (4)	−0.0034 (4)	−0.0018 (4)	−0.0025 (4)
C2	0.0306 (5)	0.0230 (4)	0.0269 (5)	−0.0050 (3)	−0.0005 (4)	−0.0003 (4)
C3	0.0302 (5)	0.0236 (4)	0.0296 (5)	0.0037 (3)	0.0035 (4)	−0.0019 (4)
C4	0.0291 (6)	0.0589 (8)	0.0333 (6)	0.0091 (5)	−0.0011 (5)	0.0093 (6)
C5	0.0330 (6)	0.0403 (6)	0.0310 (6)	−0.0095 (5)	0.0017 (5)	0.0058 (5)
C6	0.0396 (6)	0.0409 (7)	0.0371 (8)	0.0117 (5)	−0.0027 (5)	0.0067 (5)

### Geometric parameters (Å, º)

**Table:**

N1—O1	1.4218 (11)	C3—H3B	0.977 (19)
O1—H1	0.87 (2)	C3—H3A	0.93 (2)
N1—C2	1.5090 (13)	C4—H4C	0.94 (3)
N1—C1	1.5126 (14)	C4—H4B	0.987 (19)
N1—C3	1.5148 (13)	C4—H4A	1.02 (4)
C1—C4	1.5113 (19)	C5—H5C	0.96 (3)
C1—H1B	1.01 (2)	C5—H5B	0.97 (3)
C1—H1A	0.934 (18)	C5—H5A	0.91 (3)
C2—C5	1.5101 (18)	C6—H6C	0.93 (2)
C2—H2B	0.921 (19)	C6—H6B	0.93 (2)
C2—H2A	0.955 (18)	C6—H6A	1.03 (3)
C3—C6	1.5103 (19)		
			
N1—O1—H1	104.2 (17)	C6—C3—H3A	111.4 (11)
O1—N1—C2	103.23 (7)	N1—C3—H3A	104.8 (11)
O1—N1—C1	108.89 (8)	H3B—C3—H3A	110.2 (16)
C2—N1—C1	113.06 (8)	C1—C4—H4C	114.1 (16)
O1—N1—C3	109.55 (8)	C1—C4—H4B	111.0 (11)
C2—N1—C3	113.13 (8)	H4C—C4—H4B	107 (2)
C1—N1—C3	108.77 (8)	C1—C4—H4A	107.6 (18)
C4—C1—N1	113.66 (10)	H4C—C4—H4A	108 (3)
C4—C1—H1B	114.1 (13)	H4B—C4—H4A	109 (2)
N1—C1—H1B	103.7 (14)	C2—C5—H5C	111 (2)
C4—C1—H1A	111.2 (12)	C2—C5—H5B	110.9 (18)
N1—C1—H1A	106.2 (12)	H5C—C5—H5B	115 (3)
H1B—C1—H1A	107.3 (17)	C2—C5—H5A	110.5 (16)
N1—C2—C5	113.73 (9)	H5C—C5—H5A	106 (2)
N1—C2—H2B	103.4 (11)	H5B—C5—H5A	103 (2)
C5—C2—H2B	113.7 (12)	C3—C6—H6C	107.9 (16)
N1—C2—H2A	106.1 (12)	C3—C6—H6B	111.1 (16)
C5—C2—H2A	111.7 (10)	H6C—C6—H6B	108 (2)
H2B—C2—H2A	107.5 (17)	C3—C6—H6A	111.4 (14)
C6—C3—N1	113.62 (9)	H6C—C6—H6A	108.0 (19)
C6—C3—H3B	112.2 (12)	H6B—C6—H6A	111 (2)
N1—C3—H3B	104.2 (11)		
			
O1—N1—C1—C4	62.86 (11)	C3—N1—C2—C5	64.73 (12)
C2—N1—C1—C4	−51.26 (13)	O1—N1—C3—C6	−54.95 (13)
C3—N1—C1—C4	−177.82 (10)	C2—N1—C3—C6	59.62 (13)
O1—N1—C2—C5	−176.96 (9)	C1—N1—C3—C6	−173.87 (10)
C1—N1—C2—C5	−59.47 (12)		

### Hydrogen-bond geometry (Å, º)

**Table:**

*D*—H···*A*	*D*—H	H···*A*	*D*···*A*	*D*—H···*A*
O1—H1···Cl1	0.87 (2)	2.06 (2)	2.9330 (12)	175 (2)
C1—H1*A*···Cl1^i^	0.934 (18)	2.888 (19)	3.7786 (15)	159.8 (15)
C2—H2*A*···Cl1^ii^	0.955 (18)	2.943 (17)	3.6859 (16)	135.6 (14)
C3—H3*B*···Cl1	0.977 (19)	2.911 (19)	3.6203 (16)	130.3 (14)
C3—H3*A*···Cl1^i^	0.93 (2)	2.93 (2)	3.7740 (19)	150.7 (14)
C4—H4*A*···Cl1^iii^	1.02 (4)	2.98 (4)	3.9913 (18)	172 (2)
C5—H5*B*···O1^iv^	0.97 (3)	2.50 (3)	3.4359 (18)	163 (2)

Symmetry codes: (i) −*x*+1, −*y*, *z*−1/2; (ii) *x*+1/2, −*y*+1/2, *z*; (iii) −*x*+1/2, *y*+1/2, *z*−1/2; (iv) −*x*+1, −*y*+1, *z*−1/2.
